# Electrical signaling along the phloem and its physiological responses in the maize leaf

**DOI:** 10.3389/fpls.2013.00239

**Published:** 2013-07-04

**Authors:** Jörg Fromm, Mohammad-Reza Hajirezaei, Verena K. Becker, Silke Lautner

**Affiliations:** ^1^Institute for Wood Biology, Universität HamburgHamburg, Germany; ^2^Leibniz Institute of Plant Genetics and Crop Plant Research (IPK)Gatersleben, Germany

**Keywords:** action potential, assimilate translocation, callose, gas exchange, phloem, plasmodesmata, variation potential

## Abstract

To elucidate the role of electrical signaling in the phloem of maize the tips of attached leaves were stimulated by chilling and wounding. Two different signals were detected in the phloem at the middle of the leaf using the aphid stylet technique: (1) action potentials (AP) arose in the phloem after chilling; and (2) variation potentials (VPs) were evoked after wounding the leaf tip. Combined electric potential and gas exchange measurements showed that while the wound-induced VP moved rapidly towards the middle of the leaf to induce a reduction in both the net-CO_2_ uptake rate and the stomatal conductance, there was no response in the gas exchange to the cold-induced AP. To determine if electrical signaling had any impact on assimilate transport the middle of the leaf was exposed to ^14^CO_2_. Autoradiography of labeled assimilates provided evidence that phloem and intercellular transport of assimilates from mesophyll to bundle sheath cells was strongly reduced while the cold-induced AP moved through. In contrast, wound-induced VP did not inhibit assimilate translocation but did reduce the amount of the labeled assimilate in phloem and bundle sheath cells. Biochemical analysis revealed that callose content increased significantly in chilled leaves while starch increased in chilled but decreased in wounded leaves. The results led to the conclusion that different stimulation types incite characteristic phloem-transmitted electrical signals, each with a specific influence on gas exchange and assimilate transport.

## Introduction

In recent decades it has become clear that the phloem not only enables the bulk flow of assimilates but also transmits various chemical and electrical signals. Since sieve elements and their companion cells are more or less symplastically isolated from neighboring cells and contain a saline luminal fluid (80–100 mM K^+^), they represent a low-resistance channel for electrical conductance and signal transmission along the plasma membrane. It has been suggested that electrical signals play a major role in inter- and intracellular communication and in the regulation of physiological processes at both the molecular and organism level (Davies, 1987). These signals can be evoked by various stimuli, such as wounding (e.g., by chewing insects), cold-shock, heating, light-pulses or touch, and they are responsible for rapid transmission of information within the plant body in order to enable a response of distant organs to (e.g.) dangerous events. Two main types of electrical signals in plants have been identified: action potentials (APs), evoked by non-damaging stimuli, and variation potentials (VPs), triggered by mechanical damage or heating. APs are less long lasting and travel faster than VPs and are mainly transmitted along the phloem, while VPs depend on a rapid loss of tension in the xylem vessels and can travel towards the phloem to be propagated over long distances.

These two types of electrical signals are related to numerous physiological effects in plants. Insectivorous plants, for example, such as *Drosera* and *Dionaea*, use electrical signals within leaf traps as a means of catching insects in order to secure their nitrogen supply (Williams and Pickard, 1972; Hedrich, 2012). The mechanism of *Dionaea* trap closing was investigated in detail during the last years by Forterre et al. (2005) and Volkov et al. (2008), indicating that the release of elastic energy stored in the trap leaves plays a main role in the rapid closing of the trap. In *Mimosa*, meanwhile, electrical signals cause the leaflets to move, making the leaf appear unappealing to a would-be herbivore. In addition, recent studies have shown that heat-induced electrical signals cause a strong local, as well as a systemic, reduction in net CO_2_ uptake and the quantum yield of electron transport in Mimosa (Koziolek et al., 2004), poplar (Lautner et al., 2005) and maize (Grams et al., 2009).

Recently, strong evidence has been presented for a relationship between electrical long-distance signaling, Ca^2+^ influx, and cellular responses to this influx, including sieve-tube conductivity and mass flow (Van Bel et al., 2011). In the phloem of *Populus trichocarpa* the spread of a heat-induced signal depends on the availability of calcium (Lautner et al., 2005) and Ca^2+^ influx is suggested to be the key link between electrical signals and the resultant chemical responses. In maize, meanwhile, the amount of cytoplasmic calcium has been found to increase during propagation of APs evoked by cold-shock (Fromm and Bauer, 1994). Since a reduction of phloem transport was observed during electrical signaling in this earlier study of maize, the aim of the present study was to clarify the impact of phloem-transmitted electrical signals (APs and VPs) on assimilate transport and distribution within the maize leaf.

## Materials and methods

### Plant materials

Maize plants (*Zea mays* L.) were grown from seeds in pots in climate chambers under a light intensity of 400 μmol m^−2^ s^−1^ provided by mercury halide lamps and a 14 h light/10 h dark period. The temperature was 24–25°C and relative humidity was 70%. Measurements were performed on mature leaves on plants between 80 and 120 cm in height.

### Plant stimulation and sampling

While one control plant remained untreated a tip of a mature leaf of a second plant was cold-stimulated by ice water (4°C) and a leaf tip of a third plant was wounded by cutting. Electric potential recordings were made during stimulation at 8–10 cm distance from the stimulation site. For biochemical analysis the leaf tip was stimulated once per min and the middle part of the leaf was harvested in liquid nitrogen after a 15 min stimulation period. For autoradiography leaf tips were also stimulated once per min and leaves were harvested after a15 min as well as a 30 min stimulation period.

### Electric potential recordings

A mature maize leaf was excised from a plant and the cut cross-section of the leaf was submerged into a cuvette with artificial pond water (composed of 1.0 mM NaCl, 0.1 mM KCl, 0.1 mM CaCl_2_ and 1.0 mM MES, adjusted with Tris to pH 6.0) into which a reference electrode was also immersed. The leaf was placed inside a Faraday cage and aphids (*Rhopalosiphum padi*) were allowed to settle overnight on the lower side. On the following day one aphid was cut from its stylet by using a laser beam generator (Beck, Neu-Isenburg, Germany) connected to a Zeiss microscope. By using a micromanipulator the exudate at the stylet stump was then brought into contact with the tip of a microelectrode filled with 100 mM KCl. The microelectrode was connected to a preamplifier with an input impedance of >10^12^ ohms, to which an amplifier (Model 750, WPI, USA) was attached. Prior to each experiment, the microelectrode was calibrated by dipping it into a trough with artificial pond water, which was connected electrically with the reference electrode in the cuvette by an agar bridge. After connecting the microelectrode to the stylet stump, the resting potential of the sieve element was established and the tip of the leaf was stimulated at 8–10 cm distance from the site of the microelectrode. Action potentials were evoked by ice water (4°C) while VPs were generated by wounding (cutting) the leaf tip.

### Leaf gas exchange measurements

Assessment of leaf gas exchange was performed using a Waltz CQP-130 porometer (Effeltrich, Germany). Measuring conditions were 250 μmol m^−2^ s^−1^ light intensity, a leaf temperature of 25°C, and 60% relative air humidity.

### Autoradiography

For macroautoradiographic demonstration of phloem transport the middle of different treated leaves was exposed to 2.9 Mbq ^14^CO_2_. After 15 as well as 30 min all leaves were quickly frozen in dry-ice, freeze-dried and exposed on X-ray film. Microautoradiographs were made from the ^14^CO_2_ exposed blades inside plexiglas boxes at the middle of the leaves after 15 min exposure. Small sections of the leaves (1–3 mm in diameter) were quickly frozen in isopentane which was pre-cooled with liquid nitrogen. After freeze drying and embedding in Spurr's resin medium (Spurr, 1969) sections were cut with a Reichert Ultracut E microtome and fixed with 0.1 N NaOH and 5% H_5_IO_6_, which later improved the staining with 0.05% toluidine blue, pH 7.0. The sections were coated with liquefied photoemulsion (Ilford L4), exposed for 6 weeks and developed in a Kodak D-19 developer.

### Biochemical analysis

After the different treated leaves were quickly frozen in liquid nitrogen, phosphorylated intermediates were measured according to Hajirezaei et al. (2006). ATP was assayed in 100 mM Tris-HCl (pH 8.1), 0.25 mM NADP^+^, 0.85 mM Glc, 0.56 units of Glc-6-P dehydrogenase, and 0.7 units of phosphoglucose isomerase. 0.6 units of hexokinase were added to start the reaction. Other glycolytic intermediates and soluble carbohydrates were analysed as described by Stitt et al. (1989).

Starch measurement was carried out essentially as described by Ahkami et al. (2009). The sediment from the extraction was washed two times in 0.5 ml of ethanol and suspended in 0.8 ml of 0.2 M KOH. After incubation for 1 h at 95°C, the suspension was neutralized by 0.14 ml of acetic acid and centrifuged. Then, 45 μl of amyloglucosidase buffer was added to 5 μl of the suspension, which was incubated overnight at 37°C. The produced glucose was assayed photometrically.

Callose was measured according to the method described by Koehle et al. (1985) with some modifications. In particular, 50 mg fresh tissue was frozen after the treatment and homogenized using a mill (Retsch MM400, Germany) for 1 min in frozen state. The homogenate was mixed with 500 μl 1N NaOH and incubated for 15 min at 80°C while shaking at 100 rpm. Subsequently the homogenate was centrifuged at 14,000 rpm (425 g). 200 μl of the supernatant was mixed with 400 μl 0.1% aniline blue (Sigma-Aldrich, Germany) giving a red-violet colour. As blank 200 μl NaOH was used instead of sample. Thereafter, 210 μl of 1N HCl was added to all samples (colour turns to deep blue). The pH of all samples was adjusted by the addition of 590 μl of a mixture of 1M glycine and 1M NaOH. All samples were incubated then for 20 min at 50°C and allowed thereafter to stand for 30 min at room temperature (colour disappears). The measurement was carried out immediately after incubation using a fluorometer infinite M200 (Tecan, Germany) with an excitation wavelength at 400 nm and an emission wavelength at 510 nm.

## Results

### Electrical signaling

**Figure 1:**
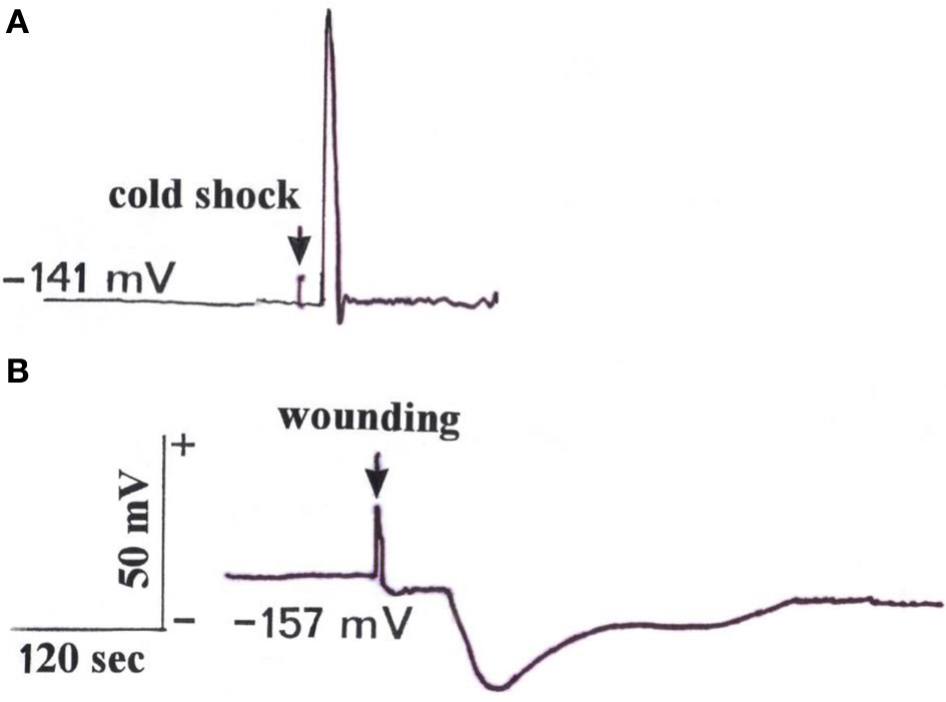
**Electrical signals measured with the aphid stylet technique in a sieve element after stimulation of the leaf tip at a distance of 10 cm. (A)** Stimulation of the tip by water ice triggered an action potential travelling with a speed of 3 cm s^−1^ in a basipetal direction. **(B)** Wounding by cutting the tip induced a variation potential propagating basipetally at a speed of 0.5 cm s^−1^. (Typical signal time courses out of a total of five measurements).

After a microelectrode was brought into contact with a cut aphid stylet, a resting potential of the sieve elements in the range of −141 to −157 mV (*n* = 5) could be detected. This resting potential was similar to the sieve tube potential of *Mimosa pudica* (Fromm, 1991). Subsequently, when the leaf tip was stimulated by water ice at a distance of 10 cm, an action potential with an amplitude of more than 70 mV and a speed of 3 cm s^−1^ was released (Figure 1A). For comparison, when the leaf tip was wounded by cutting, a VP with a short transient depolarization followed by a longer hyperpolarization, and a speed of 0.5 cm s^−1^, was detected in the phloem (Figure 1B). Regarding refractory periods, it was found that it was not possible to generate a second signal for 50 s after the first had occurred. We could detect further signals when the leaf was stimulated once per minute, however.

### Response of gas exchange to electrical signaling

**Figure 2:**
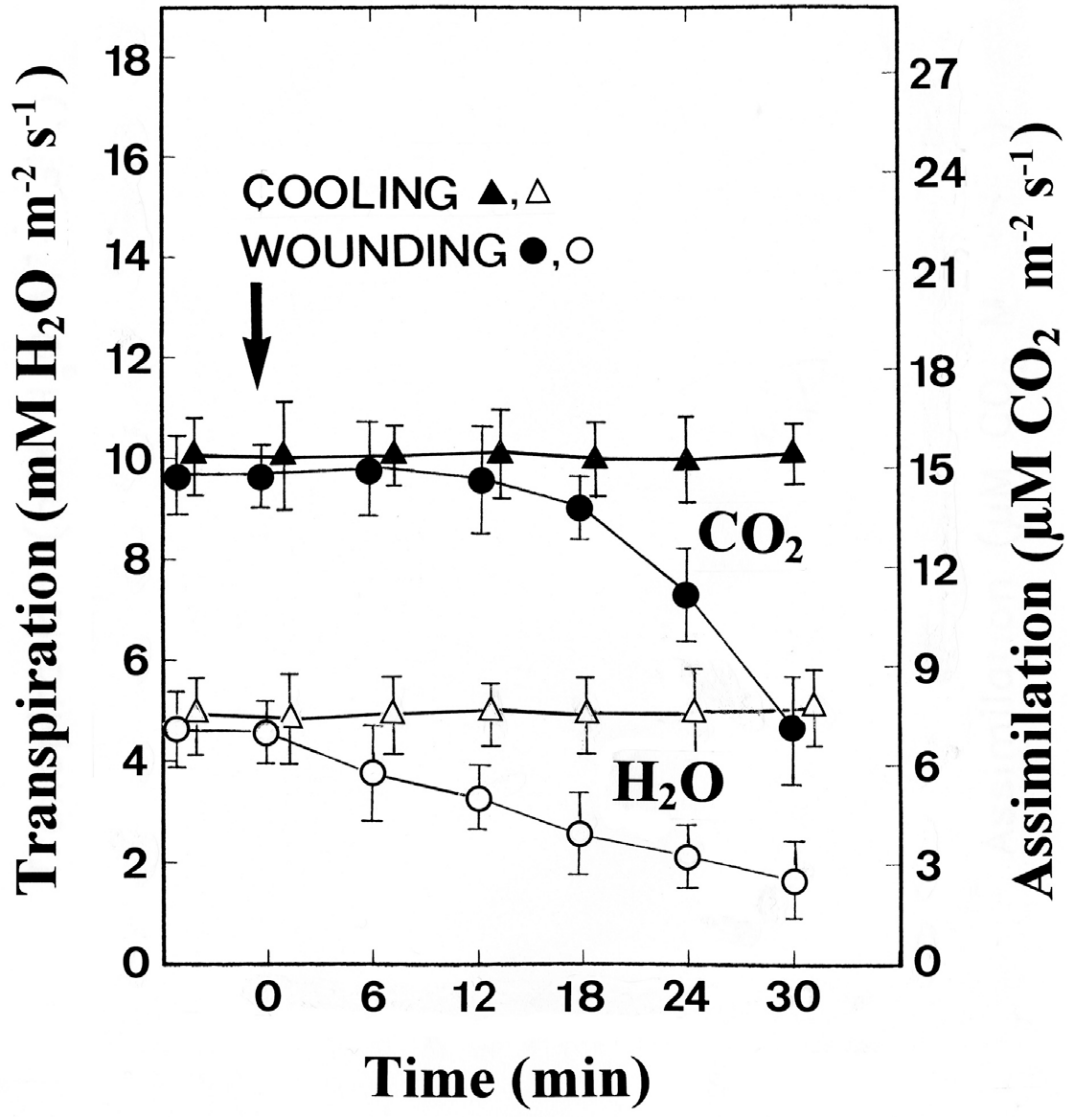
**Changes in the assimilation (CO_2_) and transpiration (H_2_O) rate after stimulation of the leaf tips by cooling or wounding**. Data are mean values ± SE from 10 leaves.

The gas exchange was measured at the middle of the leaf while the tip was being stimulated at a distance of 10 cm. Whereas cooling had no effect on both the CO_2_ assimilation and transpiration rates, wounding led to a clear reduction in both parameters (Figure 2). The transpiration rate began to decrease as soon as 6 min after wounding, indicating that wounding the leaf tip causes stomatal closure in the rest of the leaf. Simultaneously, a significant reduction in the assimilation rate was detected.

### Response of assimilate translocation to electrical signaling

**Figure 3:**
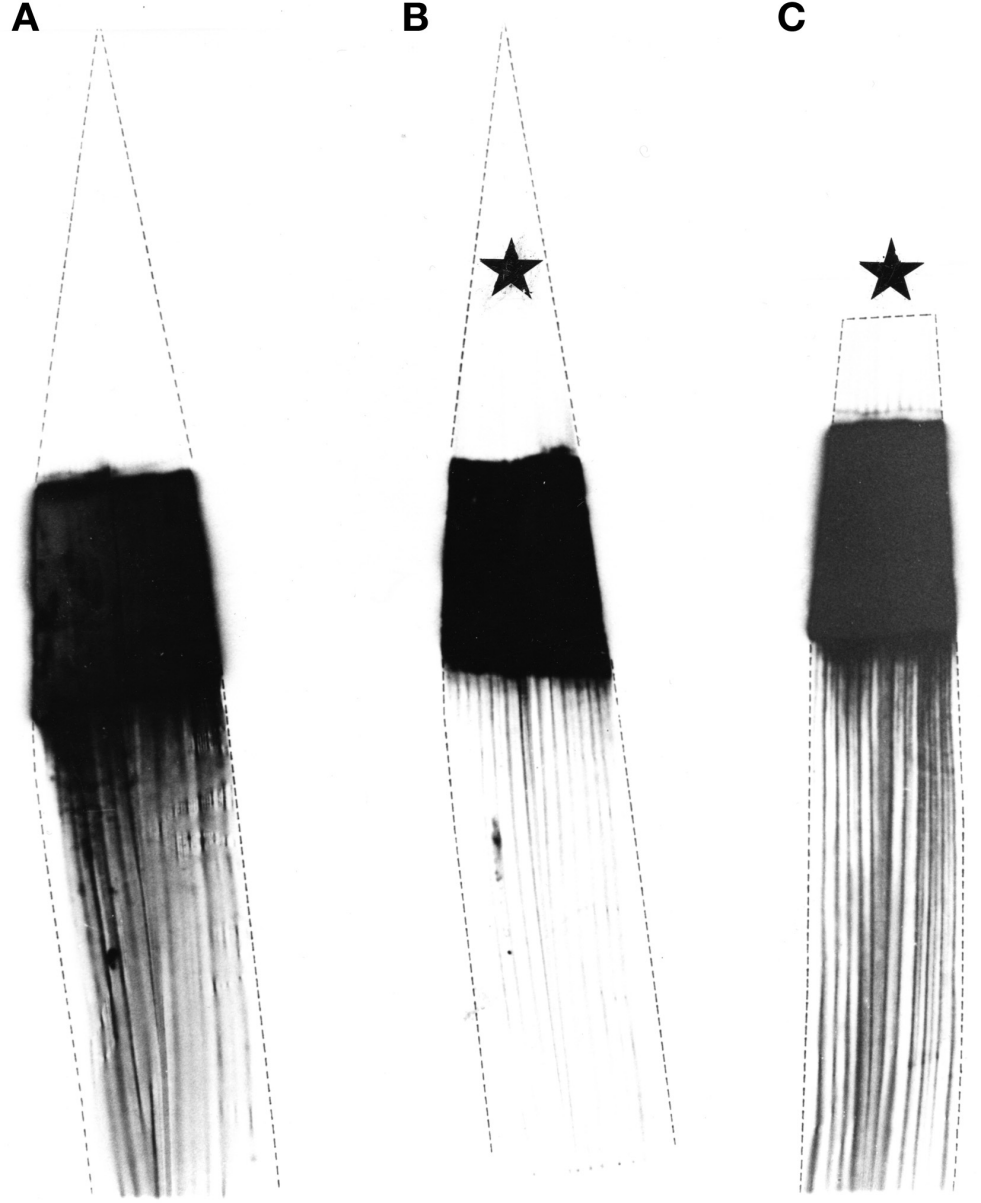
**Macroautoradiographs of three maize leaves which were labeled with ^14^CO_2_ for 30 min in the middle section**. Labeled assimilates were exported in a basipetal direction. **(A)** The left leaf remained unstimulated, **(B)** the middle leaf was cold-shocked at the tip (starlet) and **(C)** the right leaf was wounded at its tip (starlet) once per min.

**Figure 4:**
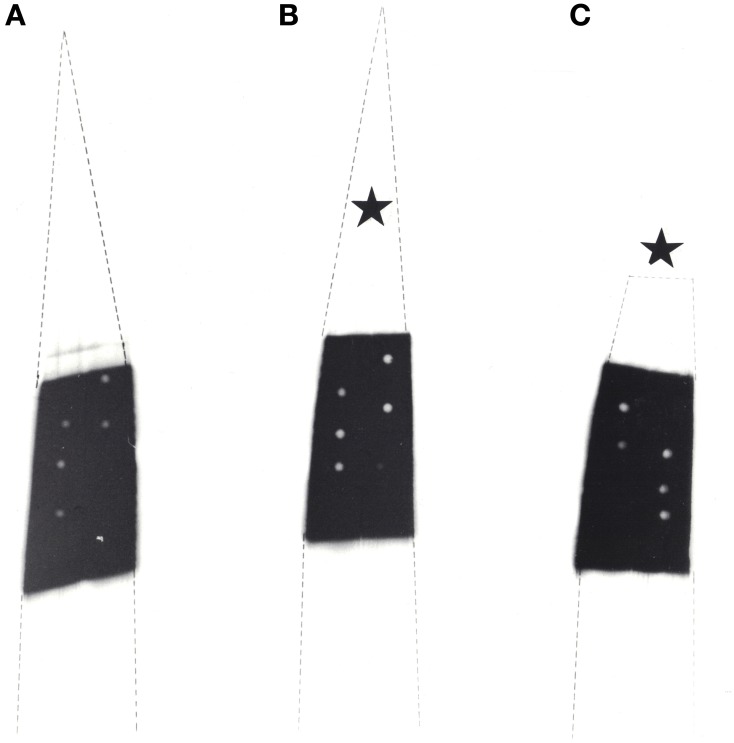
**Macroautoradiographs of three maize leaves which were labeled with ^14^CO_2_ for 15 min in the middle section. (A)** Unstimulated control leaf, **(B)** cold-shocked leaf at the tip (starlet) and **(C)** wounded leaf at the tip (starlet). The leaves were stimulated once per min. At the white circles within the labeled leaf blades samples were taken for microautoradiography.

Plants identical in height and age were predarkened for 48 h before the middle part of mature attached leaves was exposed to ^14^CO_2_ for 30 min. During this period one plant remained unstimulated while the leaf tip of a second plant was cold-stimulated (4°C) once per minute and the tip of a third plant was wounded once per minute. Macroautoradiography showed that in untreated (Figure 3A) and wounded leaves (Figure 3C) the ^14^C-labeled photoassimilates extended in the expected normal distribution from the exposed middle area (black) towards the leaf base. In contrast, in cold-shocked leaves phloem transport decreased significantly in all veins of the leaf (Figure 3B), as has previously been shown by Fromm and Bauer (1994). In another set of experiments plants were exposed to ^14^CO_2_ only for 15 min. Macroautoradiographs of such leaves showed that phloem transport did not extend into the non-exposed leaf base, either in unstimulated nor in stimulated plants (Figure 4).

**Figure 5:**
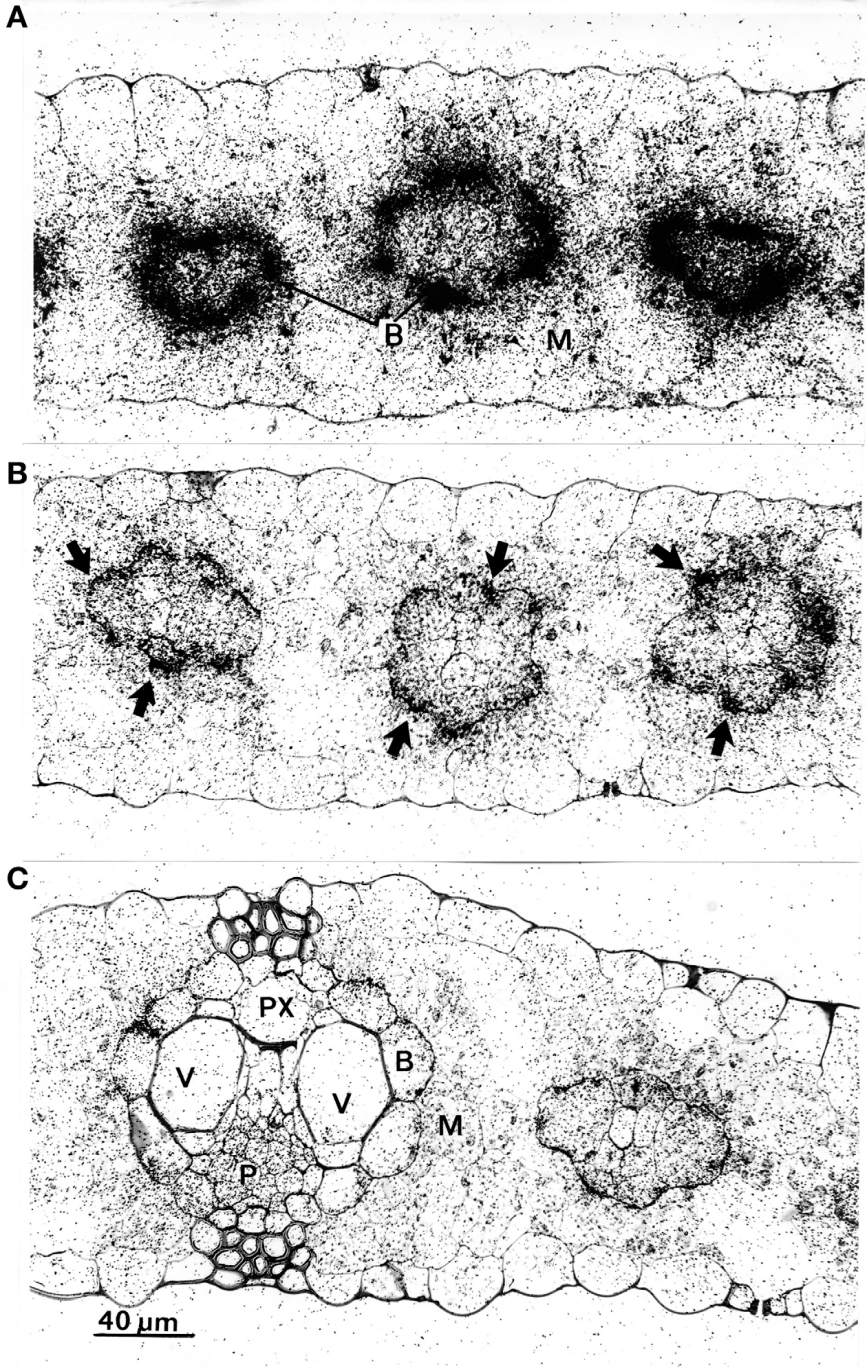
**Microautoradiographs of leaf areas which were exposed to ^14^CO_2_. (A)** In the unstimulated leaf, label is concentrated in bundle sheath cells **(B)**. **(B)** In leaves which were cold-shocked at their tips label concentrated at the cell walls between mesophyll (M) and bundle sheath cells (arrows). **(C)** In tip-wounded leaves label was more or less distributed equally in all living cells. There was a slight accumulation, however, at the mesophyll/bundle sheath cell wall. P, phloem; PX, protoxylem; V, vessel.

To check for physiological causes for the reduction of phloem transport, microautoradiographs were made of the leaf areas exposed to ^14^CO_2_. After a 30 min exposure period the tissue was heavily labeled (not shown), therefore microautoradiographs were made from leaves exposed to ^14^CO_2_ after 15 min. The results are shown in Figure 5, and reveal that in unstimulated leaves (Figure 5A) label accumulated massively in bundle-sheath cells (B) of all veins, while the mesophyll (M) was only slightly labeled. In comparison to unstimulated leaves, the microautoradiographs of cold-stimulated leaves revealed that the translocation of ^14^C-assimilates from mesophyll to bundle-sheath cells was significantly reduced (Figure 5B). Here, label appeared to be more concentrated in the outer walls of the bundle-sheath (arrows) than inside these cells. Clearly, therefore, cold-induced action potentials yielded distinct inhibition of intracellular transport in the maize leaf. The ^14^CO_2_ exposed areas of wounded leaves, meanwhile, showing some label slightly accumulating at the bundle sheath cell wall but that most of the label was distributed more or less equally in all cells of the leaves (Figure 5C).

### Biochemical responses

To check if the different patterns of labeling are related to changes in the photosynthetic metabolism, the concentrations of various metabolites in the ^14^CO_2_ exposed areas were measured photometrically after a stimulation period of 15 min. In Table 1 it is shown that, compared to unstimulated leaves, cold-shocked leaves had higher malate, PEP, PGA, TrioseP and ATP, while starch levels increased more than two-fold. Wounded leaves, in contrast, exhibited lower levels of almost all investigated metabolites. The dramatic increase of starch within the chloroplasts of the cold-shocked leaves reflects the elimination of photosynthate transport via plasmodesmata, while starch in the wounded leaves decreased clearly in correlation to the reduction in CO_2_ uptake (Figure 2).

**Table 1:** **Metabolite levels in unstimulated, cold-shocked and wounded maize leaves (*N* = 5 ± *SD*)**.

**Metabolite**	**Content in unstimulated leaves**	**Content in cold-shocked leaves [μmol g^−1^ fresh wt]**	**Content in wounded leaves**
Malate	2.10 ± 0.25	2.89 ± 0.18^*^	1.70 ± 0.45
PEP	0.35 ± 0.05	0.38 ± 0.17	0.27 ± 0.13
Pyruvate	0.54 ± 0.29	0.45 ± 0.15	0.29 ± 0.11
PGA	1.52 ± 0.38	1.61 ± 0.50	0.94 ± 0.16
DHAP	0.68 ± 0.12	1.10 ± 0.17^*^	0.32 ± 0.13^*^
GAP	0.04 ± 0.01	0.24 ± 0.05^*^	0.13 ± 0.01^*^
Sucrose	0.99 ± 0.18	0.86 ± 0.10	1.35 ± 0.21
Glucose	0.20 ± 0.07	0.18 ± 0.05	0.17 ± 0.09
Fructose	0.08 ± 0.01	0.08 ± 0.01	0.08 ± 0.02
ATP	0.13 ± 0.02	0.17 ± 0.05	0.11 ± 0.04
Starch	1564 ± 398	3640 ± 1050^*^	1040 ± 86

(Asterisks indicate significant differences to control—P = 0.05—, calculated by student's t-test). The photometrically measured data show that cold-shocked leaves have higher malate, PEP, PGA, TrioseP, ATP and starch levels than untreated leaves, whereas wounded leaves have lower levels of almost all metabolites.

**Figure 6:**
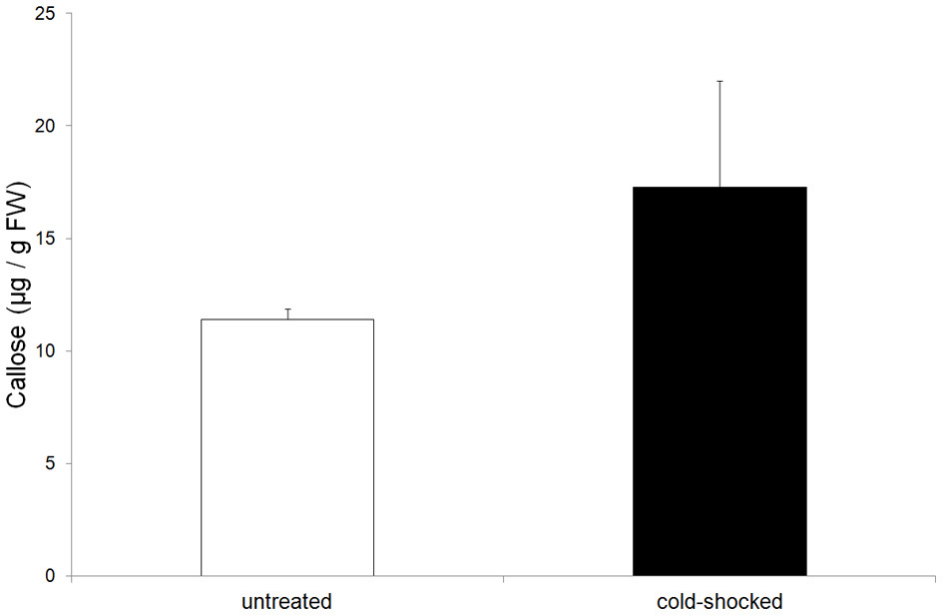
**Concentration of callose in untreated and cold-shocked leaves (*n* = 4 ± SD)**. Callose increased significantly in cold-shocked leaves.

Callose is well known as a substance which serves to plug plasmodesmata and occlude sieve-plate pores. Measurements showed that in cold-shocked leaves callose concentrations increased significantly compared to the unstimulated control leaves (Figure 6). Obviously, the movement of photoassimilates from the mesophyll cells to the bundle sheath cells would be inhibited by closed plasmodesmata in cold-stimulated leaves.

## Discussion

Previous studies on electrical signaling in maize have shown that chilling leaf tips generates action potentials with amplitudes of more than 50 mV which are transmitted basipetally in sieve tubes (Fromm and Bauer, 1994). They were measured via severed aphid stylets, as first described for the membrane potential by Wright and Fisher (1981). APs are known to be generated by potassium, chloride and calcium fluxes, and to reduce phloem transport in maize. In addition to cold-shock induced action potentials, re-irrigation of drought-stressed maize plants also induce action potentials, travelling via the phloem from roots to leaves in order to regulate photosynthesis (Grams et al., 2007). Apart from cold and watering stimuli, heat stimulation also evokes electrical signals in maize that travel through the leaf while reducing the net CO_2_ uptake rate and the photochemical quantum yield of both photosystems I and II (Grams et al., 2009).

The controlling factors in the reduction of phloem transport caused by cold-induced action potentials are so far not clear, however. On the one hand, it is well known that chilling localized areas of the transport path inhibits phloem transport in many plant species, due to a mechanical blockage of sieve plate pores or to a change in the hydrostatic pressure and osmolarity of the sieve sap. Additionally, abrupt drops in temperature are also known to cause an interruption of assimilate transport in plants (Pickard et al., 1978; Minchin and Thorpe, 1983). The fact that cold-induced action potentials also inhibit phloem transport distant from the stimulation site, and the mechanisms through which this is achieved still need to be clarified, however, and was one aim of the present study. In this regard, callose is well known as a substance that serves to plug plasmodesmata and occlude sieve-plate pores. In regard to sieve-plate occlusion there is evidence that this is a dual process involving protein in tandem with callose, since sieve elements have been observed to occlude quicker by proteins than by callose production (Furch et al., 2007, 2010). In *Vicia faba*, dependent on a rise in calcium concentration, protein bodies, so-called forisomes (Knoblauch et al., 2001), disperse abruptly coincident with the propagation of an electrical signal (Furch et al., 2007). In intact *Cucurbita maxima* plants, however, a different mechanism for occlusion has been found: sieve element proteins coagulate several centimeters away from the stimulation site, while several minutes after stimulation callose deposition reached its maximum (Furch et al., 2010). Such a dual occlusion process guarantees very rapid and effective sieve tube sealing after damage and should prevent sap loss from sieve tubes.

In the present study, microautoradiographs from the ^14^CO_2_-exposed leaf areas showed that label accumulated at the outer walls of bundle sheath cells upon cold stimulation (Figure 5B). Furthermore, the levels of malate, PEP, TrioseP and ATP increased. These metabolites occur primarily in the mesophyll of maize leaves and their increase indicates that photosynthate transport via plasmodesmata is interrupted. This conclusion is confirmed by two other observations:

- the movement of photosynthetic intermediates between mesophyll and sheath cells is restricted to the plasmodesmata (Evert et al., 1977) and;
- a clear increase in the callose level in cold-shocked leaves (Figure 6).

The increase in callose level is most likely to be responsible for occluded plasmodesmata and thus an interruption in the intracellular transport of photosynthetic intermediates which might also lead to a reduction of the phloem loading process. Furthermore, electrical signaling via plasmodesmata is a well-known phenomenon (Van Bel and Ehlers, 2005) and plasmodesmata provide a route for the passage of electric current between cells (Spanswick and Costerton, 1967; Spanswick, 1972). The macroautoradiographic demonstration of reduced phloem transport in cold-shocked leaves (Figure 3B), therefore, might be caused by a decrease of phloem loading as well as by callose-occluded sieve-plate pores. A possible link between electrical signaling and callose biosynthesis might be the calcium-induced activation of callose synthase. Since action potentials are known to be based on an initial influx of calcium (Kikuyama and Tazawa, 1983; Beilby, 1984) an increase in cytoplasmic calcium during electrical signaling might also serve to activate callose synthase. Apart from callose, starch also increased significantly in cold-shocked leaves (Table 1), probably due to the interruption of assimilate transport and the fact that the CO_2_-uptake rate does not respond to cold-induced action potentials (Figure 2).

In contrast to chilled leaves, tip-wounded leaves exhibited a decrease in both CO_2_ uptake and transpiration rate (Figure 2). The transpiration rate began to decrease as soon as 6 min after wounding and was followed by a significant reduction in the assimilation rate. These observations are in close agreement with the reduction of label in the cells of the ^14^CO_2_ exposed leaf areas (Figure 5C) and indicate that wounding the leaf tip causes stomatal closure and photosynthetic decline in the middle of the leaf. Consequently, starch content decreased in wounded leaves in comparison to the unstimulated control leaves (Table 1). When the leaf tip is wounded, the water status of the whole leaf changes and thus xylem tension is lost. The resulting hydraulic wave can be transduced into ion flux changes through mechanosensory channels in the neighboring living cells (Stankovic et al., 1998; Davies and Stankovic, 2006) to generate a VP which moves laterally via the plasmodesmata into the phloem from where it can be propagated over long distances (Figure 1B). VPs (also called slow wave potentials because of their slow repolarization phase) are therefore a likely candidate for transmitting the wounding stimulus and have been extensively studied in numerous plant species such as cucumber and pea seedlings (Stahlberg and Cosgrove, 1992, 1994). Regarding the ionic mechanism, VPs usually are initiated by mechanosensitive Ca^2+^ channels and also involve a transient shutdown of a P-type H^+^-ATPase in the plasma membrane (Stahlberg et al., 2006). However, in maize wounding by cutting triggered a VP with a short transient depolarization followed by a long-lasting hyperpolarization (Figure 1B). The short depolarization exhibits AP-like kinetics which also has been reported after repeated flaming of sunflower leaves (Davies et al., 1991; Stahlberg et al., 2006). Since it is unlikely that the hyperpolarization is caused by a shutdown of the proton pump, in future experiments other ionic fluxes are needed to explain signals of the negative sign, such as K^+^-efflux or an activation of the proton pump.

In conclusion, in a previous study (Fromm and Bauer, 1994) we provide evidence that cold-shock induced action potentials spread via the phloem through the leaf, and upon signal transmission, phloem transport is reduced. In the present study a range of evidence is given that an increasing callose level occludes the plasmodesmata between mesophyll and bundle sheath cells. As a consequence, phloem loading is reduced and metabolite levels increase within the mesophyll. In a similar way, wound-induced VPs also travel via the phloem, however, in contrast to action potentials, they lead to a reduction in photosynthesis and callose content. Future studies will be directed towards a better understanding of the relationship between electrical signals and physiological responses within the veins.

### Conflict of interest statement

The authors declare that the research was conducted in the absence of any commercial or financial relationships that could be construed as a potential conflict of interest.
